# Adaptive Significance of Non-coding RNAs: Insights from Cancer Biology

**DOI:** 10.1093/molbev/msae269

**Published:** 2025-01-07

**Authors:** John F McDonald

**Affiliations:** Professor Emeritus, School of Biological Sciences, Integrated Cancer Research Center, Georgia Institute of Technology, Atlanta, GA, USA

**Keywords:** evolution, cancer, adaptation, non-coding RNAs, transgenerational inheritance

## Abstract

The molecular basis of adaptive evolution and cancer progression are both complex processes that share many striking similarities. The potential adaptive significance of environmentally-induced epigenetic changes is currently an area of great interest in both evolutionary and cancer biology. In the field of cancer biology intense effort has been focused on the contribution of stress-induced non-coding RNAs (ncRNAs) in the activation of epigenetic changes associated with elevated mutation rates and the acquisition of environmentally adaptive traits. Examples of this process are presented and combined with more recent findings demonstrating that stress-induced ncRNAs are transferable from somatic to germline cells leading to cross-generational inheritance of acquired adaptive traits. The fact that ncRNAs have been implicated in the transient adaptive response of various plants and animals to environmental stress is consistent with findings in cancer biology. Based on these collective observations, a general model as well as specific and testable hypotheses are proposed on how transient ncRNA-mediated adaptive responses may facilitate the transition to long-term biological adaptation in both cancer and evolution.

## Introduction

It has been long maintained, and is now widely accepted, that cancer biology has much to learn from the field of evolutionary biology (e.g. Merlo et al. 2006; Zhu et al. 2021). On the molecular level, both disciplines focus on the causal basis of inherited changes that may subsequently be operated on by natural selection to increase the fitness of the biological entities inheriting the changes. This process is typically associated with biological adaptation, i.e. modifications in biological entities that improve their suitability for existence in their environment. Admittedly, inherited changes that are adaptive for cancer cells are typically anything but from the perspective of cancer patients. Nevertheless, it is now widely recognized that the underlying dynamics of the adaptive process are similar in evolution and cancer.

The ultimate source of inherited biological change, whether evolution or cancer, has traditionally been associated with a broad spectrum of events on the DNA level encompassing changes in the sequence of the DNA molecule itself (e.g. nucleotide substitutions, insertions or deletions) and changes in chromosome structure (e.g. chromosomal inversions, translocations, insertion and deletions). In more recent years, another class of events (epigenetics) has been shown to involve heritable changes in chromatin configuration mediated by a variety of processes not related to those falling under the traditional definition of mutation. Regardless of cause, such inherited changes may result in differences in morphological, physiological and behavioral features (i.e. phenotypes) that may or may not be of adaptive significance. Over time, these events may result in the accumulation of heritable variation among individuals within populations (or cells within a developing tumor) that may subsequently be operated on by selection.

Cancers are generally believed to initiate from a single variant “pre-cancer” cell. The number of descendants derived from this initial abnormal cell will expand and accumulate new heritable variation over time until the descendant cells are identifiable as a “polyclone” of abnormal cells and, if they continue to progress, are eventually characterized as a primary tumor. Variants that increase the replicative efficiency (or fitness) of descendant cancer cells are referred to by cancer biologists as cancer “driver mutations”. These genetic variants are comparable to what are called “adaptive mutations” by evolutionary biologists. At the other end of the spectrum, those genetic variants referred to by evolutionary biologists as adaptively “neutral mutations” that are maintained in populations through non-selective mechanisms (e.g. drift and/or linkage to selected genes) are what cancer biologists call “passenger mutations”.

Adaptive responses to environmental challenges, whether in cancer or in natural populations (or polyclones), may be transient or long term. Transient adaptive responses have been traditionally considered to be physiologically based and non-heritable (e.g. “phenotypic plasticity”). Long-term adaptive responses, in contrast, are generally considered to be mutationally based and associated with the traditional Darwinian paradigm of selection operating on standing genetic variation present in populations. Mutation is widely recognized as the ultimate source of heritable genetic variation. However, the relative importance of the mutation process as a rate limiting step in adaptive evolution has long been the subject of debate and depends upon the relative levels of potentially adaptive (or “driver”) genetic variation present in the environmentally challenged population.

The relationship between transient and long-term adaptive evolutionary responses, especially with respect to the issue of whether the former can somehow transition into the later, has been debated by evolutionists from at least the time of Lamarck. This debate has been resurrected in recent years, centering around the issue of the potential evolutionary significance of environmentally-induced epigenetic changes that may be inherited across generations. Evolutionary biologists have recognized the potential adaptive significance of inherited epigenetic changes for several years (Jablonka 2017; Loison 2021) and several mathematical models have more recently been developed demonstrating the potential evolutionary impact of these changes (e.g. Bonduriansky and Day 2009; Day and Bonduriansky 2011; Klironomos et al. 2013; Banta and Richards 2018). For example, Geoghegan and Spencer (2012) extended previous models on phenotypic plasticity by Kirkpatrick and Lande (1989) to integrate epigenetically encoded plastic responses into population-epigenetic models of selection.

Interestingly, the potential adaptive significance of inherited environmentally-induced epigenetic changes is an area of high interest in the field of cancer biology as well. In cancer biology considerable research has been devoted toward understanding the molecular mechanisms underlying the process. Much current focus is on the role played by non-coding RNAs (ncRNAs) in the induction of heritable epigenetic changes (including DNA methylation, histone modifications, etc.) involved in the acquisition and/or transmission of acquired adaptive traits during tumor progression. Recent findings in cancer biology related to this question may be of relevance to the topic of adaptive evolution in general.

The purpose of this review is to demonstrate how recent findings on the epigenetic mechanisms underlying the inheritance of adaptive changes over cancer onset and development may provide insight into mechanisms underlying environmentally-induced epigenetic changes in adaptive evolution. Toward this end, I first present detailed examples of the epigenetic mechanisms underlying the adaptive response of cancer cells to a variety of environmental challenges. I next summarize recent findings indicating how these transiently adaptive epigenetic responses may be transformed into stably inherited changes across generations. Finally, I summarize preliminary evidence that the ncRNA-mediated epigenetic mechanisms underlying the biological adaptation of cancer cells to environmental stress are operating generally in both plants and animals and conclude by proposing a model on how epigenetic responses to environmental stress may contribute generally to adaptive evolution.

### The Adaptive Significance of Changes in Gene Regulation

Early studies on the molecular basis of both evolution and cancer were almost exclusively focused on mutations in genes encoding proteins and, more specifically, on those single nucleotide mutations (substitutions, insertions, deletions) that can alter a protein's amino acid sequence. However, it was soon realized by both evolutionary (e.g. Britten and Davidson 1969; McDonald 1983) and cancer biologists (e.g. Morange 1997) that genetic variation regulating the expression of genes (either cis- or trans-) can also be of adaptive significance. Indeed, the importance of such regulatory changes in both evolution and cancer remain today areas of active investigation (e.g. Ferlier and Coulouarn 2022; Vaishnav et al. 2022).

Most regulatory changes that have thus far been shown to contribute to the adaptive process in both evolution and cancer fall well within the Darwinian paradigm of random mutation followed by selection. Nucleotide level changes and the insertion or deletion of transposable elements in the promoter or enhancer regions of genes (i.e. cis-regulatory mutations), can affect a gene's level of expression. Likewise, when such mutational events occur within the cis-regulatory or coding regions of genes encoding transcription factors or other regulatory RNAs or proteins, they may induce significant changes in the expression of their gene targets (trans-regulatory mutations). In both instances, such regulatory variants may be subsequently operated on by selection consistent with established and well-documented Darwinian processes. In recent years, however, there has been intense interest in the potential evolutionary significance of heritable regulatory changes not associated with changes in DNA sequence (e.g. Ashe et al. 2021; Yi 2024).

### Epigenetic-Mediated Regulatory Change

The term “epigenetics” was initially defined by the developmental biologist C.D. Waddington in 1942 as “the causal interactions between genes and their products, which bring the phenotype into being” (Van Speybroeck 2002; Waddington 2021). The contemporary use of the term has been much expanded and encompasses a variety of molecular processes affecting heritable changes in gene function that are associated with DNA but NOT with changes in DNA sequence or structure per se (Hamilton 2011). A key characteristic that distinguishes epigenetic regulatory changes from other types of regulatory controls is the fact that epigenetic regulatory changes can be environmentally induced and subsequently inherited across cell divisions.

The inheritance of epigenetic-mediated changes in gene expression across somatic cell divisions was first recognized by developmental biologists as a critical component of the establishment of tissue-specific patterns of gene expression during embryogenesis (Felsenfeld 2014). The first empirical indication that epigenetic changes may also be playing a significant role in the onset and progression of cancer was the observations that the promoter regions of many tumor suppressor genes in newly established cancer cells are heavily methylated and that these epigenetic marks are typically transmitted to progeny cells (e.g. Baylin and Jones 2016).

A primary mechanism contributing to the passage of epigenetic information across cell divisions is mediated by a family of enzymes called DNA methyltransferases (DNMTs; Denis et al. 2011). A newly synthesized unmethylated DNA strand is produced each replication cycle and DNMTs recognize hemi-methylated target sites and appropriately methylates the newly synthesized strand thus maintaining the epigenetic signal across cell divisions (Hermann et al. 2004). Conversely, the regulated loss of DNA methylation patterns is mediated by another class of enzymes as exemplified by TET2 (tet methylcytosine dioxygenase 2) in humans (e.g. Chen et al. 2020).

The methylation and demethylation of DNA sequences may also induce transient modulations in chromatin structure that, in turn, trans-regulate the accessibility of various types of regulatory proteins and RNAs to DNA thereby further influencing gene expression. Another class of enzymes contributing to the passage of epigenetic information across cell divisions are histone acetyltransferases/deacetylases (HATs/HDACs). These enzymes can install or remove acetyl groups on lysine residues of regulatory proteins such as histones, transcription factors, and nuclear receptors, thereby regulating gene expression (Kouzarides 1999). It is now known that many ncRNAs play a major role in regulating all these epigenetic enzymes and in the epigenetic process in general (Kaikkonen et al. 2011).

**Fig. 1.**
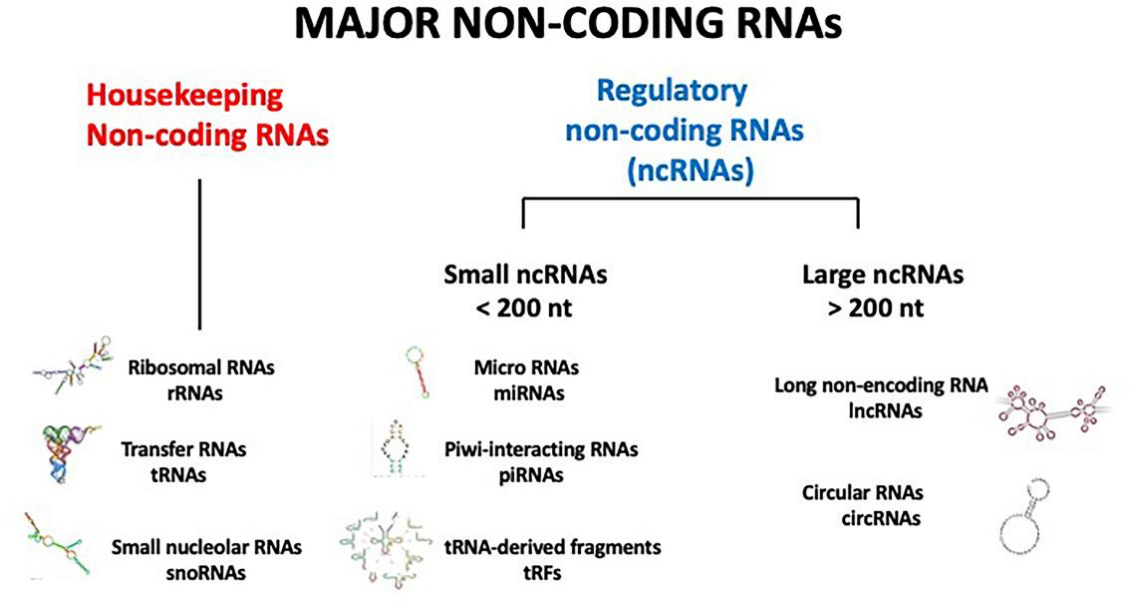
NcRNAs play critical roles in eukaryotic housekeeping and gene regulatory functions. Small ncRNAs are typically <200 nt in length and encompass sub-categories including tRFs, miRNAs, piRNAs and snoRNAs. LncRNAs and circRNAs are >200 nt in length and are comprised of RNA polymerase I (Pol I)-, Pol II-, or Pol III-transcribed RNAs and are often derived from processed introns. All of these RNAs control a variety of regulatory functions that contribute significantly to cancer onset/progression.

Originally described, along with transposable elements, as “junk DNA”, regions of eukaryotic genomes encoding untranslated RNAs (e.g. >90% of the human genome is transcribed but not translated) are now known to play important roles in eukaryotic housekeeping functions (rRNAs, tRNAs and snoRNAs) or gene regulation. The regulatory ncRNAs [henceforth referred to simply as ncRNAs] involved in various aspects of genome regulation are numerous and diverse (Fig. 1). Small ncRNAs (sncRNAs) are typically <200 nucleotides (nt) in length and encompass a rapidly growing list of sub-categories including tRNA-derived fragments (tRFs), microRNAs (miRNAs), piwi-interacting RNAs (piRNAs), and small nucleolar RNAs (snoRNAs). All of these contribute significantly to eukaryotic gene regulation and, perhaps not unexpectedly, to cancer onset and progression as well (Uchida and Bolli 2018). A second class of ncRNAs, long non-coding RNAs (lncRNAs), and circular RNAs (circRNAs) are >200 nt in length and are comprised of RNA polymerase I (Pol I)-, Pol II- or Pol III-transcribed RNAs and are often derived from processed introns. These longer RNAs can interact with a variety of RNA-binding proteins (RBPs) to assemble ribonucleoprotein (RNP) complexes (Liu et al. 2022) that have been associated with a vast array of regulatory functions including mRNA splicing, mRNA stability and genetic imprinting (Uchida and Bolli 2018). Interestingly, it has been found that the same lncRNA may be associated with transcriptional activation or repression depending upon the specific RBP with which it is complexed (Long et al. 2017).

Among the most extensively studied and thus, at this point, the most well-understood class of ncRNAs are miRNAs (O’Brien et al. 2018). MiRNAs can regulate target genes at both the transcriptional and post-transcriptional levels. These small ncRNAs regulate gene expression of targeted genes post-transcriptionally by inhibiting the translation of gene transcripts (mRNAs) and/or by degrading gene transcripts (mRNAs) prior to translation. Interestingly, most miRNA post-transcriptional regulation involves direct interaction (typically RNA–RNA pairing) with targeted mRNAs within the context of a multiprotein complex called RISC (RNA-induced silencing complex).

MiRNAs themselves may be post-transcriptionally regulated by circRNAs and lncRNAs that may serve as a class of competitively binding endogenous RNAs (ceRNAs) thereby preventing miRNAs from interacting with their primary gene (mRNA) targets within the RISC. Indeed, the regulatory potential of lncRNAs extends well beyond their capacity to serve as ceRNAs. LncRNAs can also serve as scaffolds in the recruitment of, e.g. DNA methyltransferases, resulting in the targeted DNA methylation of various cancer driver genes across generations (Huang et al. 2022). They may also directly complex with histone acetyltransferases that, as mentioned above, can install acetyl groups onto lysine residues of regulatory proteins such as histones, transcription factors, and nuclear receptors, thereby regulating cancer driver gene expression.

The full spectrum of the molecular functions of ncRNAs are varied and complex and range from epigenetic transcriptional level regulation (both cis- and trans-) to post-transcriptional regulation of the translational process. Details of these processes have been extensively described elsewhere (e.g. Hombach and Kretz 2016; Wei et al. 2017) and are beyond the scope of this review.

## Responses of Cancer Cells to Environmental Stress Mediated by ncRNAs

In this section, we will consider the role played by ncRNAs in the adaptive epigenetic response of cancer cells to various environmental stresses. The focus here is on understanding the molecular mechanisms underlying this adaptive response. Because cancer cells are somatic cells, acquired epigenetic information is passed to descendent cells mitotically during tumor development. In following sections of this review, we will consider the mechanisms by which this acquired epigenetic information can be transferred from somatic cells to germline cells.

### The Immune Response

When cancer cells arise, they are often recognized as “foreign” within the context of the organism in which they appear and, consequently, they typically elicit an immune response. This will usually result in destruction of the nascent cancer cells unless blocked by a rapid and effective adaptive response. For instance, normal cells produce molecules, called “checkpoint inhibitors” (e.g. PD-L1) that help prevent destruction of normal cells by immune cells (T-cells). Cancer cells recognized as “foreign” by the immune system and initially targeted for destruction will often adapt by overexpressing PD-L1 and other checkpoint inhibitors to, in effect, mask themselves from the immune response thereby avoiding destruction. The mechanisms underlying this rapid adaptive response are complex but have been demonstrated to critically involve the stress-induced expression of circRNAs and lncRNAs. These subsequently bind to and thus inhibit the action of specific miRNAs involved in the modulated control of PD-L1 and other check point proteins in normal cells (e.g. Xu et al. 2020; Zhang et al. 2020).

It is important to keep in mind that any environmentally-induced ncRNA-mediated adaptive response, such as exemplified above, is often complemented or replaced at later stages of cancer development by selection for cells or clones carrying genetic variants that provide the same adaptive phenotype [e.g. regulatory mutations that increase the expression of PD-L1 (e.g. Stein et al. 2021)].

While the role played by the immune system in destroying “foreign” cells (e.g. pathogens, aberrant cancer cells, etc.) is widely recognized, often overlooked is the fact that the immune system is also involved in tissue repair, regeneration and tissue remodeling (Laurent et al. 2017). Thus, although the immune system is certainly capable of destroying cancer cells, it is also capable of contributing to their survival and proliferation depending on the microenvironmental context. Cancer cells exploit this dual capacity of the immune system by not only over-producing immune-inhibiting signaling molecules (e.g. check point proteins like PD-L1) that block their destruction but by producing signals that stimulate the immune system's potential to foster cell proliferation as well. This latter adaptive strategy has also been shown to be regulated by ncRNAs (e.g. Zhang et al. 2020).

To give an example, the most abundant type of immune cells in the tumor microenvironment are tumor-associated macrophages (TAMs). TAMs display two alternative phenotypes. M1 macrophages are primarily involved in promoting the immune system's inflammatory responses that may include the destruction of cancer cells. In contrast, M2 macrophages promote the anti-inflammatory and pro-growth responses of the immune system. It is now well-documented that ncRNAs produced by cancer cells (e.g. miR-375 and miR-21; Frank et al. 2019), are transported to TAMs via exosomes [i.e. extracellular vesicles (ECVs) produced by the challenged cells]. This transfer induces formation of the M2 phenotype thereby enhancing cancer cell growth/proliferation (Shinohara et al. 2017; Hsieh et al. 2018; Cao et al. 2019; Frank et al. 2019; Jiang and Li 2022).

### The Oxygen Deprivation Response

In addition to the ncRNA-mediated adaptive response of cancer cells to the immune system, ncRNA-mediated adaptive responses of cancer cells to a variety of other microenvironmental challenges are also well documented. The limited vasculature and supply of red blood cells coupled with the elevated metabolic demands associated with rapid cell proliferation present cancer cells with the serious challenge of reduced oxygen availability. A variety of ncRNAs (miRNAs, lncRNAs, circRNAs and piRNAs) have been associated with the adaptive response of cancer cells to oxygen deprivation (a.k.a., hypoxia; Chang et al. 2016; Barreca et al. 2021).

A family of transcription factors called the hypoxia-inducible factors (HIFs) has long been known to play a central role in the activation of a variety of genes involved in the biological adaptation of cancer cells to oxygen deprivation (Choudhry and Harris 2018). In non-hypoxic conditions, the expression of the *HIF* genes is repressed in normal cells by miRNAs (Joshi et al. 2014; Krutilina et al. 2014; Wu et al. 2015) that become downregulated in cancer cells in response to hypoxia (typically mediated through interactions with circRNAs and/or lncRNAs; Cantile et al. 2021; Peng et al. 2021; Lai et al. 2022). This down-regulation of miRNAs results in the upregulation of the HIF transcription factors (e.g. Hif-1α) that, in turn, activate genes involved in the increased production of oxygen-bearing red blood cells (erythropoiesis; Semenza 2022). In non-hypoxic environments miR-217 targets and represses the HIF encoding gene *HIF-1*α. In low oxygen environments, the lncRNA HOTAIR is expressed and serves as a ceRNA that binds to miR-217 thereby de-repressing *HIF-1*α and elevating levels of HIF in cancer cells (Hong et al. 2017).

An additional adaptive response of cancer cells to oxygen deprivation involves the upregulation of miRNAs leading to the increased delivery of oxygen-rich blood to cancer cells through the formation of new blood vessels (angiogenesis; e.g. Kim et al. 2014). In addition to miRNAs, hypoxia-induced expression of many lncRNAs and circRNAs has also been implicated in the adaptive response of cancer cells to oxidative distress (Peng et al. 2021; Lai et al. 2022) either through direct epigenetic interactions with HIF and associated regulatory genes or indirectly by the inhibition of miRNAs involved in the repression of the hypoxia response (Cantile et al. 2021).

Interestingly, a number of mutations have been identified in several different advanced- stage cancers that mimic the ncRNA-mediated adaptive responses of cancer cells to hypoxic conditions. As was the case for the ncRNA-mediated transient immune response, some of these heritable mutations map to genes directly involved in the HIF response (e.g. Krieg et al. 2000) while others map to upstream regulators of *HIF* gene expression (e.g. Ravi et al. 2000; Zundel et al. 2000). Thus, the adaptive response of cancer cells to oxygen deprivation often involves both selection for appropriate genetic variants as well as environmentally-induced ncRNA-mediated response.

### The Nutrient Deprivation Response

The energetic demands of the rapid division of cancer cells results in a significant depletion of glucose levels that, in turn, induces a ncRNA-mediated adaptive response. Glucose deprivation in cancer cells induces expression of the lncRNA, *Linco156* that subsequently serves as a ceRNA to sequester miRNA inhibitors (miR107/103a-3p) of the phosphoglycerate dehydrogenase (*PHGDH*) gene. This silencing leads to PHGDH-mediated activation of the serine synthesis pathway that, in turn, supports cancer cell survival and proliferation in glucose depleted environments (Zhang et al. 2021).

Significant increases in the expression of PHGDH are also characteristic of many advanced cancers and have been associated with heritable mutations (typically structural amplification) of the *PHGDH* gene (Mullarky et al. 2011) or loss-of-function mutations in upstream repressors of PHGDH expression (e.g. p53; Rathore et al. 2020). Thus, as was the case in the adaptive response of cancer cells to oxygen deprivation, the ncRNA-mediated transient response of cancer cells to glucose depletion may be complemented or replaced over cancer development by selection for adaptive genetic variants as well.

Similar scenarios linking ncRNAs with the rapid adaptive response of cancer cells to other types of intrinsic microenvironmental stress typically associated with tumor progression (e.g. acidosis, endoplasmic reticulum stress, etc.) are equally well documented (Lv et al. 2022). As was the case for the specific responses outlined above, these ncRNA-mediated adaptive responses are often complemented or replaced at more advanced stages of the disease with selection for functionally equivalent adaptive genetic variants when they are available.

### Adaptive Response of Cancer Cells to Therapeutic Agents

In addition to the various intrinsic challenges faced by cancer cells during tumor development, cancer therapy constitutes an additional level of environmental challenge to cancer cells requiring an adaptive response. Again, ncRNAs are significant players in this process.

A common first line defense that cancer cells employ against chemotherapeutic drugs is the ability to rapidly remove these toxic substances from the challenged cancer cells. The ATP-binding cassette (ABC) transporters are a family of transporter proteins that are normally responsible for the translocation (import and export) of a vast array of substances across cell membranes (e.g. the import of various nutrients, biosynthetic precursors, trace metals and vitamins) and for the export of a large variety of primary and secondary metabolites. The ABC transporters have also been shown to be frequently involved in the active removal of chemotherapeutic drugs from cancer cells (e.g. Vasiliou et al. 2009).

The levels of ABC transporters in normal cells are highly regulated. The ABC transporter ABCB1 is downregulated by miR-136 in normal cells. The transient resistance of cancer cells to the drug oxaliplatin is the result of drug-induced upregulation of the BLACAT1 lncRNA that, in turn, serves as an inhibitory sponge (ceRNA) of miR-136 leading to elevated expression of ABCB1 and export of the drug from the cancer cells (Wu et al. 2018). A variety of other molecular strategies may be taken by cancer cells in the transient acquisition of drug resistance (e.g. inhibition of the programmed cell death/apoptosis, alterations in cell metabolism, etc.) and each of these alternative paths have also been associated, either directly or indirectly, with regulatory changes mediated by ncRNAs (e.g. Peng et al. 2020; Wu et al. 2020a, 2020b).

As was the case for other microenvironmental challenges faced by cancer cells, the ncRNA-mediated adaptive response to cancer drugs (transient resistance) may be complemented by subsequent selection for genetic variants in the developing cancer clone that provide equivalent levels of resistance (intrinsic resistance; Mansoori et al. 2017). These selected genetic variants often involve the same set of genes that are activated by ncRNAs in the transient response (e.g. *ABCB1*; Skinner et al. 2023).

### Mutation Rate Responses to Environmental Stress

Thus far, we have focused on ncRNA-mediated adaptive responses of cancer cells to specific microenvironmental stresses (e.g. hypoxia, nutrient deprivation, chemotherapy). As we have seen, these responses are typically rapid and directly applicable to the environmental challenge inducing the response. In addition to these targeted responses, a more generalized response of cancer cells (and, to a greater or lesser extent, all cells) to environmental stress has been attributed to ncRNA-mediated elevation in mutation rates (e.g. Shaw and Gullerova 2021).

#### Nucleotide Mutations

It has now been widely established that cells, including cancer cells, typically display significantly higher rates of nucleotide mutation under stress conditions and that these changes are often attributable to ncRNA-mediated repression of DNA-repair systems. When a single nucleotide substitution or insertion or deletion occurs during replication it will result in a mispairing between the template (parental) DNA strand and the newly synthesized (daughter) DNA strand. These mispairings are recognized and typically corrected by the mismatch repair (MMR) pathway (Li 2008). Stress-induced repression of the MMR pathway results in a 100 to 1000-fold increase in mutation rates in eukaryotic systems (Meier et al. 2018).

One major player in the MMR pathway is the protein MSH2 (MutS homolog 2). The hypoxia-induced HIF response discussed above is associated with significant down-regulation of MSH2 expression (Koshiji et al. 2005) mediated by over-expression of the MSH2 repressor miR-155 (Bruning et al. 2011). Another strong repressor of MSH2 and other major components of the MMR pathway is the miRNA miR-21, which as we have seen above, is induced by hypoxia, and a variety of other environmental stresses (Angel et al. 2023). Interestingly, miR-21 has been found to be continually overexpressed in many advanced-stage cancers attributable to a heritable regulatory mutation controlling miR-21 expression (Volinia et al. 2006). Dysregulation of other major components of the nucleotide DNA-repair pathway in cancer includes modulation of cell cycle checkpoints (Hu et al. 2010) and the repair of double-strand breaks (Peraza-Vega et al. 2022). Both responses are under the control of stress-inducible ncRNAs [e.g. miRNAs (Moyano and Stefani 2015), piRNAs (Anwar et al. 2017) and lncRNAs (Su et al. 2018)].

As was the case for the specific ncRNA-mediated transient adaptative responses outlined previously, the ncRNA-mediated repression of nucleotide DNA-repair pathways in cancer is sometimes complemented or replaced in late-stage cancers by selection for heritable loss-of-function mutations in DNA-repair genes (e.g. Guedes et al. 2017; Jiang et al. 2020; Toss et al. 2021).

#### Transposable Element Mutations

Another highly significant class of mutations in cancer indicates those mediated by the retrotransposon family of transposable elements (e.g. Iskow et al. 2010). The movement (transposition) and subsequent insertion of retrotransposons in or near genes can result in a range of functionally significant effects ranging from loss of gene function to the creation of novel regulatory controls. The adaptive evolutionary potential of retrotransposons has long been recognized and is well documented (e.g. McDonald 1993; Miller et al. 1999; Schrader and Schmitz 2019).

Regulation of retrotransposon activity is primarily epigenetic and has been shown, in large part, to be directly or indirectly regulated by ncRNAs. LINEs (long interspersed nuclear elements) are the most abundant class of autonomous retrotransposons in the human genome. Although most LINEs in humans are no longer biologically active, there remains a subcategory of *LINE-1* elements (*L1PA1* and L1PA2) that are fully functional and capable of retrotransposition (Sheen et al. 2000; Beck et al. 2010). These *LINE-1* elements are normally repressed in both somatic and germline cells. Major players in this silencing process are piRNAs (Wu et al. 2020a, 2020b). PiRNAs interact with Piwi proteins to guide DNA methyltransferases to *LINE-1* elements resulting in their transcriptional silencing and repressed transposition. PiRNAs and Piwi proteins are also known to interact with the histone methylation machinery by regulating the methylation of histone lysine residues (H3K and H4K), which constitutes a second level of epigenetic repression regulated by piRNAs (Sugimoto et al. 2007). It is interesting to note that although *Alu* elements are the most abundant retrotransposon in the human genome (>1 million copies), they do not encode proteins themselves but rather hijack LINE-1 proteins for replication. Thus, in regulating the expression of *LINE-1* elements, piRNAs are effectively regulating *Alu* elements as well.

A variety of environmental stresses are known to undo the transcriptional repression of LINE-1 elements resulting in a significant increase in *LINE-1-* (and *Alu*)-associated mutations predominantly at early stages of tumor development when, as we have seen, nascent cancer cells encounter a variety of novel environmental challenges (e.g. Saito et al. 2010; Pisanic et al. 2019). At least some of these newly acquired mutations may be of adaptive significance for emergent cancer cells as evidenced by the fact that many of these *LINE-1* insertional mutations are fixed (at 100% frequency) in advanced-stage cancers (Rodriguez-Martin et al. 2020).

The molecular basis of the derepression of retrotransposons in response to environmental stress has been most intensively studied in *Drosophila* where it is associated with a block in the biosynthesis of piRNAs (Specchia and Bozzetti 2021) and it is believed that similar processes are at play in cancer cells as well (Goodier 2016).

### Molecular Triggers of Stress-Induced ncRNA Transcription

As we have seen, ncRNAs are directly or indirectly involved in the adaptive response of cancer cells to a variety of environmental stressors. We have also considered numerous examples of how ncRNAs can regulate themselves at the post-transcriptional level (e.g. lncRNAs and circRNAs serving as ceRNAs to regulate miRNA function) but initially there is often an environmentally-induced trigger that regulates the expression of ncRNAs at the transcriptional level. The molecular mechanisms responsible for this transcriptional activation of ncRNAs are varied and complex (Lv et al. 2022) but generally involve a cellular sensor of the stress condition that, in turn, regulates transcription factors that induce (or repress) the transcription of one or more ncRNAs. We previously alluded to the role of the HIF family of transcription factors in the activation of a variety of genes involved in the biological adaptation of cancer cells to oxygen deprivation. One member of the family, HIF-1α, when complexed with protein cofactors (HIF-1β, CBP/p300) in the nucleus, binds to hypoxia response elements (HREs) in the promoters of a series of genes encoding several ncRNAs (e.g. miR-210) involved either directly or indirectly in the adaptive response of cancer cells in hypoxic environments (Pezic et al. 2014). In non-hypoxic conditions when oxygen is abundant, –OH molecules bind to HIF-1α in the cytoplasm blocking the protein from entering the nucleus and thus repressing transcription of hypoxia-relevant ncRNAs. Interestingly, many of the hypoxia related ncRNAs induced by HIF-1α can conversely modulate HIF-1α expression, forming a positive/negative feedback loop in hypoxic stress–response (Choudhry and Mole 2016). While the molecular details underlying the transcriptional activation of cancer cell ncRNAs in various stress conditions remains an area of active investigation, the hypoxia response exemplifies the types of mechanisms currently believed to be involved (Lv et al. 2022).

## The Inheritance of Acquired ncRNA-mediated Epigenetic Information Across Generations

Innumerable studies over the last several years have confirmed the importance of various types of ncRNA-mediated epigenetic changes in nearly every aspect of cancer onset and progression including, as documented above, the inheritance across cell divisions of adaptive traits acquired by cancer cells in response to various environmental challenges. The more provocative question is whether such environmentally-induced epigenetic changes acquired in somatic cancer cells can be transferred to future generations.

The transmission of acquired traits across generations is generally classified into two categories: *intergenerational inheritance* and *transgenerational inheritance*. When germ cells (e.g. sperm or egg) acquire information encoding an environmentally-induced trait in the parental generation, it may become manifest in the mother's or father's F1 offspring and possibly in the mother's F2 generation as well (if the information was acquired in the germ cells of the fetus). On the molecular level, such phenomena would involve the mitotic transfer of the acquired epigenetic information across cell divisions. This is referred to as *intergenerational inheritance*.

In contrast, *transgenerational inheritance* is when epigenetic changes acquired in the parental generation are inherited by offspring beyond the F1 (or possibly in F2 for females if the information was acquired in the germ cells of the fetus) generations. In animals, such phenomena would require the meiotic transfer of acquired epigenetic information across multiple generations.

### The Transfer of Acquired Epigenetic Information From Somatic Cells to Germline Cells

The possible transfer of information from somatic to germline cells has long been a controversial topic because it is believed to violate long held genetic principle. August Weismann, one of the pioneers in the field of developmental biology, postulated in 1893 that hereditary information can only move from the germline to somatic cells and not vice versa (Weismann 1893). Today known as “Weismann's Barrier”, this concept maintains that environmentally induced or otherwise acquired phenotypes in somatic cells during an individual's lifetime cannot be transferred to germ cells and, thus, inherited by offspring. However, recent molecular studies have challenged the universality of “Weismann's Barrier.” Most notably, ECVs (Doyle and Wang 2019), are now known to transport small RNA fragments, peptides or metabolites (including environmentally-induced ncRNAs) not only between somatic cells but between somatic cells and germline cells as well (Chan et al. 2020; Guo et al. 2023).

### Evidence for the Inheritance of Acquired Predispositions to Cancer

It has long been established that predispositions to the development of many types of cancer can be attributable to the inheritance of DNA mutations through family lineages (Rasnic et al. 2020). However, the fact that not all predispositions to cancer can be attributed to DNA mutations led to the hypothesis that at least some predispositions to cancer may be epigenetic in origin (Baylin and Jones 2016). A large number of epidemiological studies have now clearly established that a variety of environmental factors [e.g. diet (da Cruz et al. 2020), environmental pollutants (Cohn et al. 2015; Nicolella and de Assis 2022), smoking (Ji et al. 1997), alcohol (Rompala and Homanics 2019), etc.], often associated with induced epigenetic changes, are significantly correlated with an inherited propensity of developing cancer. As suggestive as such epidemiological studies may be, they do not constitute definitive proof that acquired predispositions to cancer are transferable from parents to offspring.

A widely acknowledged barrier to the inheritance of acquired traits (including acquired predispositions for cancer) across multiple generations, at least in mammals, is the fact that all non-essential epigenetic marks (i.e. those NOT associated with essential epigenetic mechanisms such as imprinting, etc.) are erased early in development. Two main waves of demethylation occur, one at the zygote stage and the other when primordial germ cells reach the genital ridge (Shi and Wu 2009; Bollati and Baccarelli 2010). Such erasures are necessary for the zygote to be totipotent and capable of acquiring or “reprograming” tissue-specific epigenetic marks that are pre-requisite for multi-cellular differentiation during development. However, this barrier to the inheritance of acquired epigenetic information would be circumvented if the acquired information is transferred to the progeny by ncRNAs carried in gametes.

As mentioned above, it is now well documented that ECVs are capable of transferring ncRNA payloads from somatic cells to both oocytes (Guo et al. 2023) and sperm (Chan et al. 2020). Also documented above, is the fact that ncRNAs can induce epigenetic changes contributing to cancer development. The remaining question is, can environmentally-induced ncRNAs that contribute to an increased predisposition of cancer in parents be inherited by their offspring?

To date, most studies exploring the possibility of cross-generational transfer of cancer-associated ncRNAs have been focused on sperm due to technical challenges associated with conducting definitive studies in females. In addition to the simple fact that the relative abundance of oocytes available for study is far less than sperm, it is currently extremely difficult to experimentally distinguish between the potential effects of ncRNAs transferred from somatic cells to oocytes from those potentially-induced de novo in developing embryos (Kierszenbaum 2006). Such experimental ambiguities are more easily resolved when working with sperm.

Human and other mammalian sperm carry large numbers of ncRNA species including an abundance of miRNAs and tRFs (Schuster et al. 2016). As previously mentioned, environmentally-induced changes in ncRNA payloads in somatic cells are transferable to maturing sperm via ECVs subsequently resulting in significant alterations in the composition of the sperm ncRNA load (Bošković and Rando 2018). In addition, sperm ncRNAs can be delivered to oocytes at the time of fertilization and are capable of inducing signaling cascades that impact embryonic development (Kawano et al. 2012).

Direct evidence that environmentally-induced changes in the composition of ncRNAs contained in sperm can influence the propensity for developing cancer in progeny come from several recent studies carried out in model systems—predominantly rodents (e.g. Skinner and Anway 2007; Gapp et al. 2014; Chen et al. 2016; Ben Maamar et al. 2018).

A number of studies have shown that male mice exposed to poor diets or environmental toxicants or pesticides can result in multigenerational predispositions for breast cancer in female progeny (da Cruz et al. 2020). In a recent study (da Cruz et al. 2023), female progeny of male mice exposed to DDT in the pre-conception period were found to display a significantly higher susceptibility for developing aggressive breast cancers. The sperm of these DDT-exposed males were also shown to display changes in the composition of various ncRNAs relative to the sperm of genetically identical mice not exposed to the insecticide. Among the most notable changes was a significant surge in levels of miRNA-10b. Remarkably, embryonic injection of the entire sperm RNA load from the DDT- exposed males, or synthetic miRNA-10b, into unexposed embryos resulted in recapitulation of the same predisposition for breast cancer observed in the female progeny of male mice exposed to DDT.

These and similar experimental studies conducted in a variety of animal models (e.g. Rodgers et al. 2015; Lesch et al. 2019) are consistent with the above-mentioned epidemiological studies conducted in humans and generally confirm the possibility of intergenerational transfer of environmentally-induced predispositions for cancer.

### The Transgenerational Inheritance of Acquired Predispositions for Cancer Remains an Open Question

While some epidemiological studies conducted in humans and animal models appear to be consistent with the transgenerational inheritance of acquired predispositions to cancer, these findings are generally considered to be inconclusive because they are also compatible with alternative hypotheses (Horsthemke 2018). A significant barrier to the acceptance of the transgenerational hypothesis, at least in mammals, is the current lack of definitive evidence for a molecular mechanism capable of transferring acquired epigenetic information beyond the F1 (or F2 in females) generations. It should be noted, however, that this is not because of a lack of hypotheses.

As mentioned previously, the inheritance of environmentally-induced changes in DNA methylation marks across generations (potentially mediated by ncRNAs) has been generally dismissed because of well-established evidence that non-essential epigenetic marks are erased early in mammalian development. However, the recent identification of molecular mechanisms that help define which epigenetic marks are erased and which are not, suggests the possibility that some acquired methylation marks may, in principle, escape erasure in some situations (Ghai and Kader 2022). Thus far, however, there is no evidence that such a mechanism is associated with acquired predispositions for cancer.

In *C. elegans,* evidence for the transgenerational inheritance of environmentally-induced epigenetic changes mediated by ncRNAs is more well-established (Houri-Zeevi et al. 2020). The underlying mechanism has been attributed, at least in part, to the cross-generational replication of RNAs via RNA-dependent RNA polymerases (RdRPs; Rechavi et al. 2014). However, the existence of such enzymes has yet to be demonstrated in mammals (Pinzón et al. 2019).

A third possibility is that acquired RNA-mediated epigenetic information may be converted to DNA via reverse transcriptase (RT), i.e. RNA-dependent DNA polymerase, and thereafter meiotically inherited across generations. In higher eukaryotes, RT is an essential component of retrovirus (and retrotransposon) replication. The fact that ncRNAs are frequently found encapsulated into infectious retroviral particles during the replication process (Telesnitsky and Wolin 2016) raises the intriguing possibility that induced ncRNAs associated with increased predispositions to cancer may be transferrable via viral infection. Additional evidence that ncRNAs are also commonly associated with and reversed transcribed by endogenous retrotransposons (including human *Alu* elements; Matylla-Kulinska et al. 2014) substantiates the possibility of RT-mediated transgenerational transfer of acquired ncRNAs. If such RT-mediated transfers of acquired ncRNAs do occur, they would most likely be identified as new genetic variants (mutations) that may or may not be subsequently favored by natural selection. At present, there is no definitive experimental evidence that such mechanisms are contributing to the cross-generational transfer of acquired predispositions to cancer but given the well-documented association of ncRNAs with retroviral-like particles, this remains a viable possibility.

### Section Summary

The molecular basis of biological adaptation to environmental stress is a complex process that has been intensively studied within the context of cancer onset and progression. Recent findings (summarized in Table 1) indicate that ncRNAs are key regulators of the adaptive epigenetic response of cancer cells to environmental stress and suggest that similar processes may be at play in adaptive evolution.

**Table 1:** Summary of recent findings in cancer biology with potential relevance to adaptive evolution

(1) ncRNA-mediated epigenetic changes play a significant role in the transient biological adaptation of cancer cells to a variety of environmental challenges.
(2) ncRNAs (esp. lncRNAs) interact with a variety of RBPs to assemble RNP complexes that can regulate a vast array of regulatory functions. The regulatory role of specific ncRNAs may change depending upon the RBP with which it is complexed.
(3) ncRNA-mediated epigenetic changes play a significant role in the transient elevation of both nucleotide and retrotransposon-associated mutation rates.
(4) The transient ncRNA-mediated adaptive responses of cancer cells to environmental challenges are often complemented or replaced by functionally equivalent genetic variants (mutants) as tumors progress. Often these equivalent variants map to the same genes involved in the initial transient response.
(5) Induced ncRNAs can be transferred from somatic to germline cells via ECVs.
(6) ncRNAs carried by germline cells can transfer predispositions to cancer to F1/F2 progeny (intergenerational inheritance).
(7) Epidemiological evidence for the transgenerational inheritance of predispositions to cancer in humans (and mammalian experimental models) is currently inconclusive.
(8) While there are a variety of plausible molecular mechanisms that may contribute to the transgenerational inheritance of predispositions to cancer in humans (and mammalian experimental models), none have yet been experimentally validated.

## Preliminary Evidence for the Role of ncRNAs in Adaptive Evolution

When life-threatening environmental changes occur, organisms (or cells) must successfully respond (i.e. adapt) to survive. Gradual, yet significant, changes in environmental conditions may provide ample opportunity for natural selection to operate on standing genetic variation within populations or species to insure successful biological adaptation. However, when appropriate variation is not present and or when the environmental challenges are sudden and dramatic, more immediate adaptive responses may be required. The mechanisms by which organisms (or cells) respond to sudden and dramatic environmental threats are varied and will depend upon the nature of the threat (e.g. severity of the environmental change, the time span involved, etc.) as well as the resources available to the organisms (cells) under threat. Organisms (cells) that are mobile may, at least initially, seek to remove themselves from the stressful environment. When this is not possible (e.g. plants and other limited or non-mobile organisms or any organism when the stress is extensive or otherwise unavoidable), the response will be internal often requiring induced adaptive changes in gene regulation.

The potential evolutionary significance of epigenetic changes was initially recognized soon after it was determined that at least some epigenetic modifications may be transferable across generations (e.g. Jablonka and Raz 2009). In the following years, a number of computational models were developed supporting the potential evolutionary significance of epigenetic changes (e.g. Day and Bonduriansky 2011; Herman et al. 2014). In addition, several molecular hypotheses were proposed on how epigenetic changes may arise and contribute to adaptive evolution (Loison 2021). One hypothesis postulates that environmental stress may induce alternatively methylated versions of genes in gametes providing an at least transiently inherited adaptive response (Geoghegan and Spencer 2012). Another hypothesis proposes that alternatively methylated versions of genes may pre-exist (at least transiently) in natural populations thereby complementing DNA sequence variants in providing additional options for natural selection (Daxinger and Whitelaw 2012; Klironomos et al. 2013), and experimental evidence has been presented supporting such phenomena (e.g. van der Graaf et al. 2015; Stajic et al. 2019).

These prior hypotheses remain viable possibilities today but, as we have seen, recent findings in cancer biology indicate that altered patterns of DNA methylation patterns and other inherited epigenetic changes are often (if not always) directly or indirectly mediated by ncRNAs. These recent findings in no way contradict earlier evolutionary hypotheses but rather complement them by providing an underlying molecular mechanism that may, in fact, further support the potential evolutionary significance of environmentally-induced epigenetic changes. While it is generally acknowledged that environmental stress may occasionally induce epigenetic responses (e.g. methylation) in germline cells, it is also widely recognized that the vast majority of environmentally-induced adaptive epigenetic changes occur in somatic cells (Skinner and Guerrero-Bosagna 2009). The finding that stress-induced ncRNAs can be transferred from somatic to germline cells provides an added perspective on the evolutionary potential of epigenetic changes.

### The Central Role Played by ncRNAs in Cancer Adaptation is Consistent With What Has Been Observed in Other Species

Viewing cancer progression as a model of adaptive evolution has both advantages and limitations. One obvious limitation is that cancer progression is an adaptive process as it is manifest within the context of a human or mammalian disease. Thus, it is important to distinguish between adaptive processes and mechanisms that may be unique to humans or mammalian cancers from those that are likely to be generalizable across all organisms and species. As it turns out, however, many of the ncRNA-mediated adaptive epigenetic mechanisms underlying cancer progression have now been at least tentatively identified in other species and are widely recognized as being of potential evolutionary significance (Ashe et al. 2021; Kronholm 2023).

The phenomenon of environmentally-induced transient adaptation (a.k.a. “phenotypic plasticity”) has long been recognized and studied by evolutionists (e.g. Fusco and Minelli 2010; Fox et al. 2019). More recent molecular analyses on select animal and plant species have revealed that, while the specifics may differ, there are remarkable similarities across species in the epigenetic processes underlying these environmentally-induced adaptations. Essentially all epigenetic changes (e.g. DNA methylation or demethylation, histone modifications, etc.) are protein or enzyme-mediated (e.g. DNA methylases, histone acetolyses, etc.). In those biological systems where in-depth molecular analyses have been carried out, these epigenetic changes are either directly or indirectly regulated by ncRNAs (Frías-Lasserre and Villagra 2017; Liu and Zhong 2024) consistent with what is observed in cancer.

### Epigenetic Mechanisms Underlying the Intergenerational Inheritance of Predispositions to Cancer are Similar to Those Involved in the Inheritance of Acquired Adaptive Traits in Other Species

While there is general agreement concerning the importance of environmentally-induced adaptive responses of organisms to environmental stress, there has and continues to be debate among evolutionists as to whether such transient responses should be considered evolution (see e.g. Zimmer 2022). A typical objection is that because transiently-induced adaptive changes are not heritable, they cannot be subject to natural selection and thus do not contribute to phenotypic evolution.

Although it is generally accepted that genetic variants controlling the function and or expression of regulators of transient adaptive responses (e.g. genetic variants encoding or regulating the expression of enzymes such as DNA methyltransferases, etc.) may well be subject to natural selection, this is considered, by some evolutionists (see e.g. Zimmer 2022), as a far cry from the inheritance of specific environmentally-induced adaptive traits. However, as we have seen, the validity of these objections is being challenged by growing evidence that the induction of specific ncRNAs is at the basis of many transient adaptive responses in both plants and animals and that these induced ncRNAs can, in many instances, be inherited across generations.

The proximate cause of the (mitotic) inheritance of acquired epigenetic traits during cancer progression has long been associated with the acquisition of modified DNA methylation patterns or post-translational modifications of histone proteins affecting the expression of one or more cancer driver genes (e.g. Baylin and Herman 2000). As discussed above, the mitotic transfer of these epigenetic marks over cancer progression is mediated by a variety of enzymes that copy the marks from the parental DNA strand to the newly synthesized progeny DNA strand during the replication process (Hermann et al. 2004). In plants (Lucibelli et al. 2022) and at least some (non-mammalian) animals (e.g. Zebra fish; Skvortsova et al. 2019), similar mechanisms are involved in the transfer of epigenetic information across generations. The transfer of such epigenetic marks across generations is generally precluded in humans and other mammals due to the erasure of these marks during gamete development. However, in humans and other mammalian experimental models, ncRNAs induced in somatic cells are now known to be transferable to germline cells via ECVs thereby establishing a pathway for the intergenerational transfer of acquired traits. Indeed, ECVs can serve as transporters of ncRNAs not only among cells within a variety of plant (Wang and Dean 2020) and animal species (O’Brien et al. 2020) but, in some cases, across phyla as well (e.g. see: Lefebvre and Lécuyer 2017; Liu et al. 2023).

In *C. elegans*, environmentally-induced ncRNAs can be replicated by RNA-dependent RNA polymerase (RDRP) and, consequently, the transfer of ncRNA-mediated adaptive traits in this species typically extends to at least 3 to 5 generations (Alcazar et al. 2008). In humans and mammals where RDRP has not been detected, the intergenerational inheritance of environmentally-induced ncRNAs is currently believed to be limited to the F1/F2 generations.

### The Role of Natural Selection in the Transmission of Acquired Adaptive Traits Across Generations

The *sine qua non* of natural selection is adaptively significant genetic variation. A defining characteristic of cancer cells is uncontrolled growth, which on the molecular level, means loss of regulation of the cell cycle. There are many genes that are today known to either directly or indirectly regulate the cell cycle and a mutation in any one or more of these genes may contribute to the emergence of nascent cancer cells. However, as discussed above, newly emergent cancer cells typically find themselves in an extremely hostile environment to which they must immediately adapt if they are to survive. It is theoretically possible that some emergent cancer cells may carry mutations that pre-adapt them to the hostile environment in which they arise. However, this seems unlikely to be a common scenario because most mutations considered to be adaptive for newly emergent cancer cells in stressful environments are patently non-adaptive for normal cells (e.g. repression of the immune system, altered metabolism, disrupted angiogenesis, altered cell membrane transport, etc.) and, thus, would presumably be selected against in populations of non-cancer cells.

It must be recognized, however, that this does not preclude the possibility that cryptic genetic variants potentially adaptive for one or more of the stresses faced by nascent cancer cells may exist in pre-cancer cells but remain epigenetically silenced (thus avoiding negative selection) until environmentally activated or derepressed by ncRNAs. Indeed, the potential role of ncRNAs in the maintenance of cryptic genetic variation in natural populations has been previously proposed (O’Dea et al. 2016) and there is evidence that such ncRNA-mediated repression of cancer driver mutations in pre-cancer cells does, at least occasionally, occur (Huarte and Rinn 2010). Consistent with such a scenario is the fact that there is significant heterogeneity in the ability of individual tumor cells to epigenetically respond to the same environmental stress(es) (Beyes et al. 2021). Also consistent, are recent studies in *C. elegans* demonstrating that even when environmental stress treatments are tightly controlled, isogenic individuals display significant variability in the inheritance of ncRNA-mediated regulatory effects across generations (Houri-Zeevi et al. 2020). While it is not yet known whether this type of heterogeneity in the epigenetic response of cells to environmental stress occurs in all species, it seems reasonable to conclude that even for epigenetic-mediated adaptive traits, selection may well be playing a significant role.

### The Relevance of Environmentally-Induced Elevations in Mutation Rates

Just as it is generally agreed that genetic variation is the *sine qua non* of long-term adaptive evolution, so is it an accepted fact that mutation is the ultimate source of this variation. As we have seen, ncRNA-mediated repression of DNA-repair mechanisms in nascent cancer cells and the subsequent (often >1,000-fold) elevations in nucleotide level mutation rates is a typical response to environmental stress. Likewise, stress-induced ncRNA-mediated derepression of transposable element activity in nascent cancer cells is known to significantly elevate transposition rates leading to a significant increase in the frequency of functionally significant insertional mutations.

The evolutionary significance of stress-induced elevations in mutation rates, whether due to environmental changes experienced by species in their normal environment or as experienced by peripheral populations operating at the edge of a species ecological range, has long been recognized (e.g. McDonald 1983; Galhardo et al. 2007; Wei et al. 2022). The hypothesis is that elevated mutation rates induced in response to environmental stress may increase the probability of generating adaptive genetic variants that may subsequentially be favored by natural selection (e.g. McDonald 1983; Galhardo et al. 2007). Such a process would be expected to complement transient adaptive responses to environmental stress and this hypothesis is certainly consistent with the role of ncRNAs in cancer progression (evolution). Indeed, as we have seen, one of the most consistent characteristics of tumor progression is the observation that the genes transiently activated by ncRNAs that underlie specific stress-induced adaptive responses in nascent cancer cells, are often complemented or replaced by stress-induced mutations (often in these same genes) as the tumors progress. It is reasonable to expect that such ncRNA-mediated elevations in mutation rates in response to environmental stress are likely also at play in natural populations and, if so, may be of evolutionary significance.

**Fig. 2.**
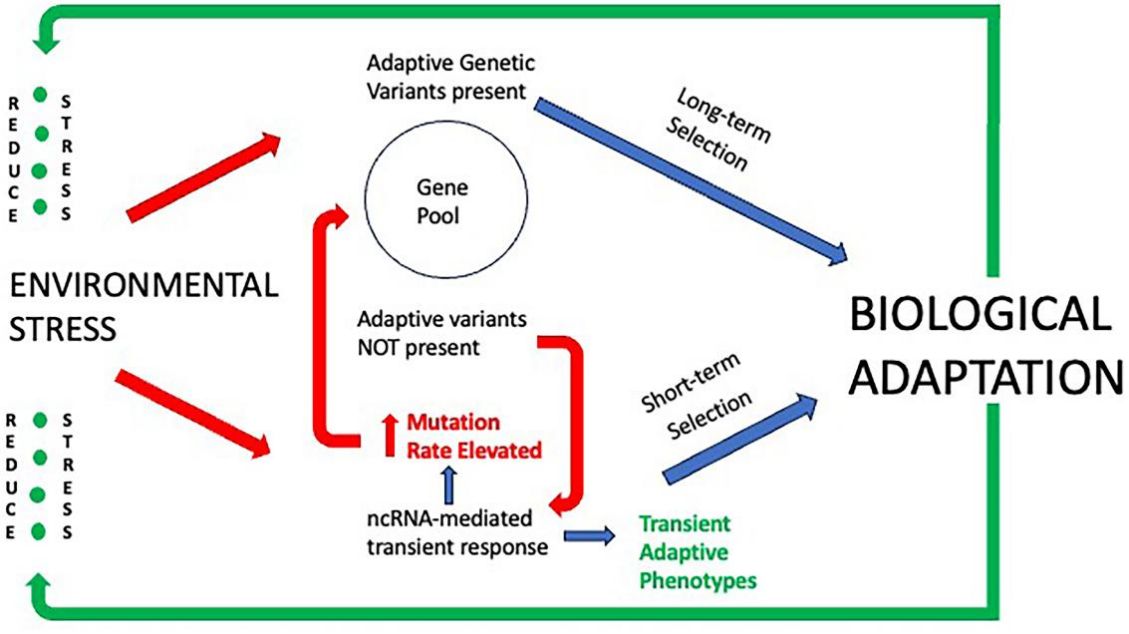
Biological adaptation to environmental stress may be transient or long term. If adaptive genetic variants are present in the gene pool or cancer polyclone of the stressed population (or nascent tumor), they will be favored by natural selection leading to long-term adaptive evolution. The fact that this is typically a time-consuming, multigenerational process has both positive and negative consequences. If the environmental stress is relatively short-term (lasting only one or a few generations), positive selection will be subsequently reduced or terminated thereby preventing premature fixation of a genetic variant that may no longer be adaptive. However, if the environmental change is rapid and substantial, the population or cancer polyclone may be unable to adapt fast enough or not at all (e.g. if pre-existing, potential adaptive genetic variants are non-existent in the challenged population/nascent tumor) thereby reducing the likelihood that the challenged population or tumor will survive. In such cases, ncRNA-mediated transient adaptive responses will be selectively favored allowing the challenged population to survive while the ncRNA-mediated elevation in mutation rates increase variability in the gene pool (polyclone) thereby increasing the probability of the existence of adaptive genetic variants favoring long-term biological adaptation if the environmental stress is prolonged or permanent.

The ncRNA-mediated activation of rapid adaptive responses to environmental stress coupled with ncRNA-mediated elevation of mutation rates would appear to constitute a highly effective pathway for the transition of transient to more permanent adaptive change (Fig. 2). Assuming the elevated number of mutations generated under stress conditions is random with respect to their potential adaptive significance, the fact that the ncRNAs responsible for the elevated mutation rates can be inherited across multiple generations would be expected to increase the probability that adaptive genetic variants will eventually emerge. However, the fact that the inheritance of ncRNA-mediated elevations in mutation rates (as well as other environmentally-induced adaptive traits) appear to be normally limited to relative few generations, provides an opportunity for a negative feedback loop that would limit the potential negative impact of stress-induced changes that are of only short-term advantage. In contrast, extended periods of environmental stress would be expected to prolong the transient elevations in mutation rate and other transient responses, thereby increasing the probability of the eventual emergence of adaptive mutations. As we have seen, just such a process is often observed in cancer progression and it is reasonable to postulate that similar scenarios may be at play in natural populations.

Of course, the number of generations required to transition from transient to heritable long-term adaptive changes would be significantly decreased if stress-induced molecular changes (e.g. stress-induced ncRNAs) could be rapidly converted to permanently heritable traits (e.g. to novel DNA variants via RT). While such Lamarckian-like mutational events have not yet been unequivocally documented even in cancer, the fact that ncRNAs are known to associate with retroviruses or retroviral particles under stress conditions (e.g. Telesnitsky and Wolin 2016) makes this an area of continuing interest in cancer biology with potentially significant evolutionary implications. It should be noted, however, that such environmentally- triggered mutations, if they do occur, would most likely be non-adaptive in those instances where the environmental stresses elicit the adaptive response are themselves transient. In such cases, environmentally-induced genetic variants would likely be selected against or possibly cryptically silenced by ncRNAs. Ongoing research in these areas will hopefully help resolve these issues in the not-too-distant future.

### Concluding Thoughts

The benefits of cross-disciplinary approaches in all areas of scientific inquiry are today widely acknowledged (e.g. Heitzmann et al. 2021). The molecular basis of cancer progression and adaptive evolution are processes that share many striking similarities (Merlo et al. 2006; Zhu et al. 2021). Indeed, the application of evolutionary principles in cancer biology is already contributing to novel approaches in cancer therapy (Enriquez-Navas et al. 2015).

The potential evolutionary significance of epigenetic changes in adaptive evolution is well recognized and the molecular mechanisms underlying these epigenetic responses are an area of active interest and investigation. The epigenetic response of cancer cells to environmental stress is an ideal system in which to pursue the underlying molecular mechanisms driving biological adaptation and recent findings have established ncRNAs as key players in the process. ncRNAs not only epigenetically regulate genes associated with transiently inherited adaptive responses of cancer cells to environmental stress, but they also transiently elevate mutation rates in response to environmental stress. The potential evolutionary significance of both processes is well known and recent findings in cancer biology strongly suggest that ncRNAs may be playing a significant role, thereby identifying this as a potentially profitable area of future evolutionary research.

Another more controversial topic in evolutionary biology is whether transiently-induced epigenetic responses can be transformed into permanently inherited traits. Recent evidence that stress-induced ncRNAs can be transferred from somatic to germline cells coupled with the observation that stress-induced ncRNAs are frequently associated with replicating retrotransposons or retroviruses (and RT) are also promising and experimentally tractable areas of future research on the molecular mechanisms underlying adaptive evolution.

## Data Availability

There are no new data associated with this article.
